# From Trait-Structured Within-Host Dynamics to SIR Models: A Multiscale Framework With Re-Exposure

**DOI:** 10.1007/s11538-026-01647-x

**Published:** 2026-05-06

**Authors:** Cyrille Kenne, Pascal Zongo

**Affiliations:** 1 Department of Mathematics, University of British Columbia, Vancouver, BC V6T 1Z2 Canada; 2 Laboratoire L3MA et IUT, Université des Antilles, Schoelcher, Martinique

**Keywords:** Trait-structured immunity, Within-host dynamics, Multiscale SIR projection, Immune thresholds, Re-exposure dynamics, 35R09, 92D25, 35Q92, 92D30

## Abstract

We present a threshold-based multiscale framework that links mechanistic within-host infection dynamics to a structured, SIR-like population model. Starting from a two-variable system for pathogen load and immune response that includes inoculum (Allee-like) thresholds and nonlinear immune activation, we derive mapping rules that classify continuous trajectories into four states: susceptible (*S*), infected with low immune protection ($I^-$), infected with high immune protection ($I^+$), and recovered (*R*). Here, “infected” refers to individuals with a detectable pathogen load. Unlike previous multiscale approaches, our framework integrates both scales into a single system: population compartments emerge by direct projection of within-host trajectories, avoiding ad hoc linking functions. We derive a next-generation operator for trait-structured re-exposure (local vs. global mixing) and an explicit expression for $\mathcal {R}_0$ under global mixing. Simulations reveal sharp clearance–persistence transitions driven by inoculum size and immune trait, and an emergent $S\!\rightarrow \!I^-\!\rightarrow \!I^+\!\rightarrow \!R$ cascade. Under sharp thresholds and activation, chronic within-host equilibria can sustain infection even when $\mathcal {R}_0<1$, producing backward-bifurcation-like behavior at the population level. The framework provides a consistent route from immunological heterogeneity to epidemic indicators, with implications for identifying chronic reservoirs, interpreting dose-response data, and estimating control thresholds directly from within-host measurements.

## Introduction

Understanding how heterogeneity in immunity shapes infection dynamics is critical for predicting both individual-level outcomes and population-scale patterns. Within-host processes such as the size of the viral inoculum, immune activation thresholds, pathogen replication rates, and the development of immune memory can vary substantially across individuals, influencing susceptibility, infectiousness, and the course of disease (Sambaturu et al. 2018; Rouse and Sehrawat 2010; Asabe et al. 2009; Guram et al. 2019). These individual differences are often encoded in measurable host traits.

Despite their biological relevance, such trait-based variations are rarely taken into account in epidemiological models, which typically rely on simplified, homogeneous representations of the host population. In standard formulations of compartmental models for infectious diseases, all relevant host properties are projected onto a small set of discrete states describing disease status, most often susceptible, infected, or recovered with respect to the progress of the disease (Anderson and May 1991). While these classical SIR-type models have proven invaluable for understanding epidemic dynamics, their homogeneous structure makes it difficult to account for mechanistic effects of immune heterogeneity observed at the individual level. To date, only a few mathematical models (see e.g., Rodrigues et al. 2009; Gomes et al. 2012; Thieme 2009a; Pérez-Reche 2025 and their references) have attempted to incorporate clinical or biological heterogeneities into population-level epidemic models, despite their known relevance at the individual level. In particular, integrating individual-level immunological information into epidemiological frameworks that explicitly account for heterogeneities in susceptibility or infectivity remains a major challenge in mathematical epidemiology (Sambaturu et al. 2018; Lloyd-Smith et al. 2015).

Classical compartmental models such as the SIR framework excel at describing how individuals flow among health states at the population scale, yet they typically omit rich features such as dose-dependent infection thresholds. Within-host models often exhibit Allee-type behavior: a minimum inoculum is required to initiate pathogen establishment (Han et al. 2019; Schmid-Hempel and Frank 2007; Van Damme et al. 2021). Responding to this need, a variety of dose-dependent models have been proposed (see the review Lunn et al. 2019). Many of these adopt pharmacokinetic-pharmacodynamic (PKPD) frameworks that couple within-host viral kinetics to dose-response relationships (e.g., Lacroix et al. 2012; González-Sales et al. 2017; Ooi et al. 2020). Other approaches include thermodynamic formulations applied to emerging viruses (Gale 2018). In addition, Xu et al. (2023) develops a detailed within-host model and couples it with dose-response functions to examine how the timing of exposure influences transmission. Despite this progress, there is still a shortage of dynamical frameworks that represent dose-response processes using explicit, biologically interpretable state variables (e.g., inoculum size, trait-dependent infection thresholds, immune activation) and that can be carried consistently into population-level models.

A separate, though related, challenge concerns how to couple within-host processes with between-host transmission. Numerous modeling approaches have emerged to tackle this coupling. A seminal example is the model by Gilchrist and Sasaki (2002), which integrates mechanistic within-host dynamics with population-level epidemiology to derive life-history trade-offs and coevolutionary outcomes. Building on this foundation, several studies have developed frameworks that further link detailed within-host processes to population-level epidemic models (see, e.g., Gilchrist and Coombs 2006; Cai et al. 2017; Gulbudak et al. 2022 and references therein). More recent multiscale frameworks track deterministic within-host progression and link it stochastically to transmission events, providing efficient numerical methods at both scales while still capturing individual transient within-host dynamics and stochasticity in transmission between hosts (Smith and Ashby 2025). However, many multiscale couplings specify transmission through an externally defined force of infection, and do not include population-level feedback directly in the within-host equations.

To address these limitations, this paper develops a single unified framework with two key novelties. First, at the within-host scale, we formulate a trait-structured model that explicitly incorporates inoculum size, trait-dependent infection thresholds, nonlinear immune activation, and re-exposure dynamics. This extends recent mechanistic approaches that emphasize inoculum effects, variability in immune thresholds, and dose-response heterogeneity by incorporating trait-dependent thresholds and re-exposure dynamics (Brouwer et al. 2017; Voutouri et al. 2023). Second, by grouping individuals according to their immune trait and their infection status (measured by the amount of pathogen they carry), we construct a new immuno-epidemiological multiscale model by naturally projecting the within-host dynamics onto a population-level system with four biologically meaningful compartments: susceptible (*S*;  undetectable pathogen load and low protection), infected with low immune protection ($I^-;$ detectable load, low protection), infected with high immune protection ($I^+;$ detectable load, high protection), and recovered (*R*;  undetectable load, high protection).

In many multiscale models, the between-host component (e.g., an SIR-type system) is constructed independently and then connected to a within-host model through a mapping from within-host variables (such as viral load) to infectiousness or transmission risk (and sometimes mortality) Childs et al. (2019); Lou et al. (2015); Kenne et al. (2023). For example, Xue et al. (2022) couple within-host viral kinetics to between-host transmission by mapping an individual’s viral load to infectiousness and embedding this in a contact-network transmission model. They also project continuous disease progression into SIR states by defining recovery when viral load falls below a given threshold, after which individuals are assumed to have lifelong immunity.

There are also multiscale models that include explicit feedback from population-level processes into individual dynamics through additional coupled mechanisms. For instance, Garira et al. (2014) couple within-host infection dynamics to between-host transmission through a pathogen-mediated feedback at the population level. More recently, Sun et al. (2025) propose a coupled within-between host framework that also includes behavioral dynamics driven by perceived risk, creating an additional pathway by which population-level state can influence transmission and infection outcomes.

Our approach here defines the epidemiological classes (*S*, $I^-$, $I^+$, *R*) emerging directly from a continuous trait-structured within-host model via a well-defined projection. While we also use threshold-based definitions to summarize within-host trajectories, we focus on how people differ in their baseline immunity and how immunity changes over time. Our model also includes a two-way feedback: infected individuals contribute to the population-level infection pressure (through shedding), and that pressure feeds back into each host’s within-host dynamics through re-exposure. So the within-host trajectory can be modified by ongoing exposure. More precisely, repeated contacts can sustain or boost pathogen load when immunity is insufficient. By incorporating a baseline immune trait $y_0$ and immune-dependent protection, the framework can represent immune waning, re-exposure, and threshold effects. In addition, the formulation of re-exposure as trait-local mixing allows us to contrast localized spread where infections propagate gradually among immunologically similar hosts with global mixing, where exposure is averaged across all traits. This distinction highlights how the structure of contacts in trait space fundamentally alters invasion thresholds and epidemic cascades.

We derive a next-generation operator for trait-structured re-exposure and obtain an explicit expression for $\mathcal {R}_0$ under global mixing, establishing the invasion threshold. Our simulations demonstrate that (i) nonlinear infection thresholds induce sharp transitions between clearance and persistence depending on inoculum size and immune trait, (ii) the emergent population-level dynamics follow a cascade $S \rightarrow I^- \rightarrow I^+ \rightarrow R$, which may not be fully captured by classical homogeneous SIR models, and (iii) backward-bifurcation-like behavior can occur: even when $\mathcal {R}_0<1$, infections may persist through chronically infected hosts who fail to clear the pathogen. These findings underscore the biological and epidemiological significance of trait-structured immunity and re-exposure in shaping epidemic trajectories.

The rest of the paper is organized as follows. In Section 2, we formulate the within-host model with re-exposure. Section 3 focuses on projecting the within-host model with re-exposure onto a between-host (epidemiological) framework. In Section 4, we derive an expression for the basic reproduction number, $\mathcal {R}_0$. Section 5 presents several scenarios. Finally, Section 7 offers discussion and concluding remarks, along with suggestions for future research directions.

## Model Formulation

We develop a novel trait-structured within-host model to explicitly capture how heterogeneity in initial immunity shapes infection dynamics and re-exposure risk. For clarity, the main variables, parameters, and functions are summarized in Table 1. Classical models typically assume a single “average” host with fixed parameters. In contrast, our framework characterizes each host by a structured initial immune trait $y_0 \in [y_{\min }, y_c]$, which determines both the initial immune competence and the infection threshold.

Biologically, $y_0$ can be viewed as a baseline level of protection present at the moment of first exposure or first inoculation. In this view, $y_0$ summarizes between-host differences in the capacity to prevent the establishment of infection (Paludan et al. 2024). Such baseline protection can reflect measurable components of Epithelial cells in barrier tissues which perform a critical immune function by detecting, restricting, and often directly eliminating extrinsic pathogens (Constant et al. 2021). It may also include measurable pre-exposure humoral immunity, since neutralizing and binding antibody levels measured prior to exposure are predictive of reduced subsequent infection risk (Gilbert et al. 2022). Finally, $y_0$ may reflect pre-existing cellular immunity, including cross-reactive memory T-cell responses observed near the time of exposure that associate with protection from infection in exposed contacts (Kundu et al. 2022).

Here, $y_c$ is a critical immune protection threshold that separates low from high protection. For each host, $y_0$ is fixed, whereas the immune response $y(t; y_0)$ evolves dynamically: it is boosted during infection before waning back toward its baseline level $y_0$ after a prolonged recovery period without re-exposure. The trait distribution across the population is given by a probability density $p(y_0)$. This formulation enables us to track how variability in baseline immunity shapes both individual trajectories and population-level dynamics.

### Remark 2.1

Many within-host models treat each individual in complete isolation, with no interaction or feedback from other hosts. Classical epidemiological models, in contrast, represent exposure only at the population scale via aggregated prevalence, without resolving individual immune and infection states. Our framework bridges these two levels by directly coupling an individual’s within-host dynamics to the shedding and immune states of others through a trait-structured re-exposure mechanism. This dual-scale coupling is a distinctive feature of our model, enabling within-host trajectories to be influenced by the evolving state of the population.

### Within-Host Dynamics

In this section, we formulate the within-host model. Its formulation is based on the following biological assumptions.

#### Assumption 2.2

*(Biological assumptions)*

- The trait $y_0$ encodes inter-individual differences arising from genetic or developmental origins, setting the baseline immune potential at infection onset.
- The immune response $y(t; y_0)$ is stimulated upon pathogen presence, with nonlinear recruitment reflecting biological activation thresholds.
- Hosts may be re-exposed via environmental contamination, with inhaled dose controlled by both the current immune state and the immunological similarity to the source hosts.

**Fig. 1:**
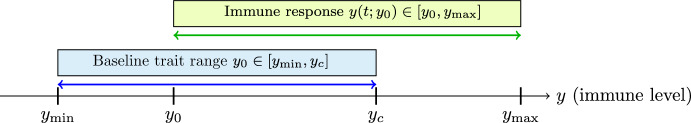
Schematic representation of immune structuring. The baseline trait $y_0 \in [y_{\min }, y_c]$ determines initial susceptibility, while the acquired immune response $y(t; y_0)$ evolves toward a maximal level $y_{\max }$ through pathogen stimulation. Here, $y_{\min }$ and $y_c$ be the minimal and the maximal baseline immune trait in the host population, respectively and $y_{\min }<y_c$.

Figure 1 shows the structuring of the host population by baseline immunity $y_0$ and the acquired immune response $y(t; y_0)$.

Let $x(t; y_0)$ be the pathogen load and $y(t; y_0)$ be the immune response of a host with trait $y_0$ at time *t*. We formulate the dynamics as:

2.1 $$\begin{aligned} {\left\{ \begin{array}{ll} \displaystyle \frac{\partial x(t ; y_0)}{\partial t} = r\,x\!\left( 1 - \frac{x}{K} \right) \!\left( \frac{x}{D(y_0)} - 1 \right) - \delta \, x\, y + \rho (t; y, y_0), \\ \displaystyle \frac{\partial y(t ; y_0)}{\partial t} = \eta \big [\, c\,(y_0 - y) + \kappa (x) \,\big ], \end{array}\right. } \end{aligned}$$

with initial conditions

$$ x(t_{y_0}; y_0) = x_0(y_0) \ge 0, \qquad y(t_{y_0}; y_0) = y_0 > 0, $$

where $t_{y_0}$ denotes the infection onset for hosts of trait $y_0$.

In the first equation of (2.1), the pathogen growth is governed by $r x\left( 1-\frac{x}{K}\right) \left( \frac{x}{D\left( y_0\right) }-1\right)$, where the factor $\left( \frac{x}{D\left( y_0\right) }-1\right)$ introduces a trait-dependent establishment threshold: when $x<D(y_0)$, net growth is negative (infection fails to establish), whereas when $x> D(y_0)$, net growth becomes positive (infection can take off). The threshold $D(y_0)$ is defined in (2.2). In the second equation, the term $-\delta x y$ represents the immune-mediated negative feedback on pathogen load: clearance increases with both pathogen abundance *x* and immune level *y*. In parallel, the immune dynamics $\frac{d y}{d t}=\eta [c(y_0-y)+\kappa (x)]$ are clarified as (i) a waning component $c(y_0-y)$ pushing the response back toward the baseline trait $y_0$, and (ii) a pathogen-stimulated recruitment term $\kappa (x)$ capturing activation upon pathogen presence. Together, these mechanisms formalize the feedback loop “higher $x \rightarrow$ higher $y \rightarrow$ stronger suppression of *x*” (and relaxation toward $y_0$). Finally, the influx term $\rho (t; y, y_0)$ is the population-level re-exposure, consistent with Assumption 2.2(c).

Infection threshold. We introduce a trait-dependent threshold

2.2 $$\begin{aligned} D(y_0) = D_{\min } + (D_{\max } - D_{\min }) \left( \frac{y_0 - y_{\min }}{y_c - y_{\min }} \right) ^n, \quad n > 0, \end{aligned}$$

which increases with $y_0$, reflecting that more immunocompetent hosts may require higher pathogen loads to establish infection (see e.g., Wu et al. 2004). The exponent *n* allows nonlinear gains in resistance, consistent with experimental observations of threshold effects in innate defense (see e.g., Hullahalli et al. 2023). Here, $D_{\min }>0$ is the minimal threshold for infection in the least immunocompetent hosts and $D_{\max } >0$ is the maximal threshold for infection in the most immunocompetent individuals (i.e., with $y_0=y_c$). Since $y_0\in [y_{\min },y_c]$, we have $\left( \frac{y_0-y_{\min }}{y_c-y_{\min }}\right) ^n\in [0,1]$. With $D_{\min }>0$ and $D_{\max }\ge D_{\min }$, it follows that $D(y_0)\in [D_{\min },D_{\max }]$, and in particular $D(y_0)>0$ for all admissible $y_0$.

Immune activation. We assume a power-law recruitment

2.3 $$\begin{aligned} \kappa (x) = \kappa _0\, x^{\ell }, \qquad \kappa _0>0,\ \ell >0, \end{aligned}$$

representing weak activation at low pathogen loads and rapid increase above a critical stimulus. This nonlinear behavior of the innate immune system is well documented (see, e.g., Gottschalk et al. 2016). This captures the sigmoidal behavior of innate immune sensors.

Re-exposure mechanism. We first define a population-driven contamination pressure experienced by a host, and then define the corresponding within-host re-exposure input after immune protection.

We introduce a population-level re-exposure pressure, defined as the contamination pressure acting on hosts at time *t*:

2.4 $$\begin{aligned} \lambda (t;y_0):=\sigma F(L_\beta (t;y_0)), \end{aligned}$$

where $\sigma >0$ denotes the rate of exposure contacts at the population level and where $L_\beta (t; y_0)$ is the effective contamination pressure experienced by a host with baseline trait $y_0$. *F* is a dose-response function of the form

$$ F(z)=\frac{d z}{b+z}, $$

where $d>0$ is the maximal dose per unit time and $b>0$ controls saturation.

Further, the within-host re-exposure input received by an individual with baseline trait $y_0$ is obtained by regulating the population-level pressure by immune protection:

2.5 $$\begin{aligned} \rho (t; y, y_0):= \lambda (t;y_0) e^{-a y(t,y_0)}=\sigma F(L_\beta (t;y_0))e^{-a y(t,y_0)}, \end{aligned}$$

where $a>0$ measures how strongly immune activity reduces uptake (or successful establishment) upon exposure. So that the multiplicative term $e^{-a y(t,y_0)}$ represents immune shielding: hosts with higher current immunity are less likely to acquire a successful reinfection.

The special case $\rho \equiv 0$ corresponds to a purely single-infection within-host model, with no secondary exposure once the initial infection is established. In this limit, individuals are completely disconnected from one another: no direct interaction through population-level contamination is possible, and the within-host dynamics evolve in isolation from the rest of the population.

Effective infection pressure. We consider two biologically motivated formulations for the effective infection pressure $L_\beta (t; y_0)$, which enters the re-exposure term $\rho (t; y, y_0)$ in (2.5) and influences the influx of pathogen load received by a host with baseline trait $y_0$.

- **Local contamination (trait-structured contacts)**:
  2.6 $$\begin{aligned} L_\beta (t; y_0) = \frac{ \int _{y_{\min }}^{y_c} w(y_0, z) \, \beta (x(t; z), y(t; z))\, p(z) \, dz }{ \int _{y_{\min }}^{y_c} w(y_0, z)\, p(z) \, dz }, \end{aligned}$$
  where $w(y_0,z)$ is a similarity kernel in trait space. This kernel can be any non-increasing function of an immunological dissimilarity $d(y_0,z)$, such as a Gaussian form $w(y_0,z)=\exp \{-d(y_0,z)^2/(2\sigma _w^2)\}$ or a compact-support kernel $w(y_0,z)=\textbf{1}_{\{d(y_0,z)\le d_{\max }\}}$. The dissimilarity *d* may represent a simple scalar gap $d(y_0,z)=|y_0-z|$ in one-dimensional models, or more biologically informed distances such as Hamming or phylogenetic distances. In this framework, the effective infection pressure on a host is driven mainly by infected individuals that are immunologically similar rather than spatially proximate. Kernel-based formulations of cross-immunity have been widely used in multi-strain epidemiology (Gog and Grenfell 2002), and remain central to linking trait-structured heterogeneity to population-level infection dynamics (Grenfell et al. 2004; Kucharski et al. 2016; Wikramaratna et al. 2015).
- **Global contamination (population-averaged contacts)**: As a simpler alternative, the effective infection pressure may be defined as a population-wide mean,
  2.7 $$\begin{aligned} L_\beta (t) = \int _{y_{\min }}^{y_c} \beta (x(t; z), y(t; z))\, p(z)\, dz, \end{aligned}$$
  corresponding to the special case $w \equiv 1$ in (2.6), which assumes that each host is exposed to the average infectious pressure generated by the whole infected population, independently of trait proximity. This mean-field formulation neglects immunological structuring, but it is analytically convenient and provides a useful benchmark when assessing the impact of trait-structured kernels.

Together, these two formulations span a spectrum of assumptions. The local kernel-based version emphasizes heterogeneity and immunological similarity, consistent with models of strain competition and cross-immunity in structured strain spaces (Gog and Grenfell 2002; Grenfell et al. 2004), while the global formulation corresponds to the well-mixed limit commonly used in population-level models (Kucharski et al. 2016; Wikramaratna et al. 2015).

Both formulations require a shedding function $\beta (x,y)$ to quantify the contribution of an infected host to environmental contamination.

#### Example 2.3

*(Shedding function)* We can adopt an immune-inhibited saturating form:

2.8 $$\begin{aligned} \beta (x, y) = \frac{\beta _0\, x}{1 + K_0\, x} \, e^{-\theta \,y}, \end{aligned}$$

where $K_0$ controls saturation at high load and $\theta$ quantifies suppression by immunity. This choice reflects that excretion capacity is finite and that immune responses reduce the viral load and the shedding (Puhach et al. 2023; Ciszewski et al. 2024). Setting $K_0=0$ yields pure exponential immune inhibition, while $\theta =0$ recovers a purely load-driven saturating shedding.

#### Remark 2.4

Standard epidemiological frameworks represent exposure at the population scale via an average force of infection depending on aggregated prevalence. Such models describe inter-host transmission but do not resolve how an individual’s within-host state evolves through explicit interaction with other hosts’ shedding levels. Some classical within-host models go to the opposite extreme, treating each host in complete isolation, which in our framework corresponds to the case $\rho \equiv 0$ where individuals are fully disconnected and no interaction through environmental contamination occurs. In contrast, our formulation (2.5)-(2.6) directly embeds trait-structured population feedback into the within-host equations, allowing re-exposure to depend explicitly on the time-varying immune and infection states of others. This unified structure links individual-scale dynamics to population-scale processes within a single mechanistic model.

**Table 1:** Summary of variables, parameters, and functions of the within-host and between-host model.

**Symbol**	**Meaning**	**Scale**
$x(t;y_0)$	Pathogen load	Within-host
$y(t;y_0)$	Immune response	Within-host
$y_0$	Baseline immune trait	Host heterogeneity
$p(y_0)$	Distribution of baseline traits	Population
$D(y_0)$	Trait-dependent infection threshold	Within-host
$\kappa (x)=\kappa _0 x^{\ell }$	Immune recruitment (power law)	Within-host
$\rho (t;y,y_0)$	Re-exposure influx	Within/Between
$L_\beta (t;y_0)$	Contamination pressure	Between-host
$\beta (x,y)$	Shedding function	Between-host
$w(y_0,z)$	Similarity kernel in trait space	Between-host
$\sigma _w$	Trait-width of local mixing kernel	Between-host
*r*	Pathogen growth rate	Within-host
*K*	Pathogen carrying capacity	Within-host
$\delta$	Immune clearance rate of pathogen	Within-host
$\eta$	Time-scale parameter for immune dynamics	Within-host
*c*	Relaxation toward baseline immunity $y_0$	Within-host
$\kappa _0$	Recruitment strength in $\kappa (x)$	Within-host
$\ell$	Recruitment exponent in $\kappa (x)$ ($>0$)	Within-host
*a*	Immune shielding coefficient	Within-host
$K_0$	Saturation parameter for shedding	Within-host
$\theta$	Suppression of shedding by immunity	Within-host
$\varepsilon$	Minimal detectable pathogen load (infection threshold)	Within-host
$y_c$	Critical immune protection threshold (low vs. high immunity)	Within-host
$\sigma$	Re-exposure intensity	Between-host
*d*	Scaling factor for contamination	Between-host
*b*	Half-saturation constant in re-exposure	Between-host
$\beta _0$	Baseline shedding rate	Between-host

### Well-Posedness of the Model

We now state regularity and boundedness assumptions ensuring that (2.1) admits a unique, biologically meaningful solution. We consider the following assumptions.

#### Assumption 2.5

(i) The function $x_0(\cdot )$ is non-negative and belongs to $C([y_{\min }, y_c])$;
(ii) The function $w(\cdot ,\cdot )$ is positive and belongs to $L^\infty ((y_{\min }, y_c)\times (y_{\min }, y_c))$;
(iii) The function $D(\cdot )$ belongs to $C([y_{\min }, y_c])$ and is uniformly bounded, i.e., there exist $D_{\min }, D_{\max }>0$ such that $D_{\min }\le D(y_0)\le D_{\max }$ for all $y_0\in [y_{\min }, y_c]$;
(iv) The function $p(\cdot )$ is the probability density function of the baseline immune trait $y_0$ in the population and satisfies $p(y_0)\ge 0$ for all $y_0\in \left[ y_{\min }, y_c\right]$ and $\displaystyle \int _{y_{\min }}^{y_c}p(y_0)\,dy_0=1$.
(v) The shedding function $\beta \in C^1(\mathbb {R}\times \mathbb {R})$ is nonnegative and satisfies $\beta (0,z)=0$, $\partial _y \beta (0,z)=0$ for all $z\in \mathbb {R}$.

Under Assumption 2.5, we can show that the model (2.1) is well posed in the sense that it admits a unique, continuously differentiable solution $(x(t; y_0), y(t; y_0))$ for all $t\ge t_{y_0}$ and $y_0\in [y_{\min },y_c]$.

#### Remark 2.6

Condition (v) has a biological interpretation: $\beta (0, z)=0$ means that in the absence of pathogens there is nothing to shed, and $\partial _y\beta (0, z)=0$ ensures that at zero pathogen load, immunity does not affect shedding even infinitesimally. This formalizes the idea that immunity influences shedding only when pathogens are present. We can easily verify that $\beta$ defined in (2.8) satisfies this condition.

In the next section, we describe how the within-host model can be projected onto a set of epidemiological compartments. This step allows us to connect individual immune-pathogen dynamics to population-level indicators, paving the way for the computation of threshold quantities such as the basic reproduction number $\mathcal {R}_0$.

## Projection of Model (2.1) onto Epidemiological Compartments

In this section, we show that the within-host re-exposure model (2.1) naturally projects onto population-level epidemiological compartments. The idea is to classify each individual, based on their current state $(x(t; y_0), y(t; y_0))$ which is a solution of model (2.1). We project each individual onto one of the compartments defined by biologically meaningful thresholds. Let $\varepsilon >0$ denote a minimal detectable or clinically relevant pathogen load (the level of pathogen at which a host is considered infected, which depends on the pathogen and diagnostic sensitivity). We recall the definition of $y_c$, a critical immune protection threshold that separates low from high protection; its value can vary depending on the pathogen and host context (see Figure 1). Although the exact values of these thresholds may be debated and context-dependent, we adopt the following classification throughout this paper.

A host is considered *Susceptible* (S) if the pathogen load remains negligible and immune protection is low, i.e., $x(t; y_0) < \varepsilon$ and $y(t; y_0) \le y_c$. If the pathogen is cleared while the immune level is high, the host is classified as *Recovered* (R), corresponding to $x(t; y_0) < \varepsilon$ and $y(t; y_0) > y_c$. Infected hosts with a significant pathogen load but weak immune protection ($x(t; y_0) \ge \varepsilon$ and $y(t; y_0) \le y_c$) are denoted as *infected with low immunity* ($I^-$), whereas those who exhibit both high pathogen levels and strong immune response ($x(t; y_0) \ge \varepsilon$ and $y(t; y_0) > y_c$) are labelled *infected with high immunity* ($I^+$).

**Fig. 2:**
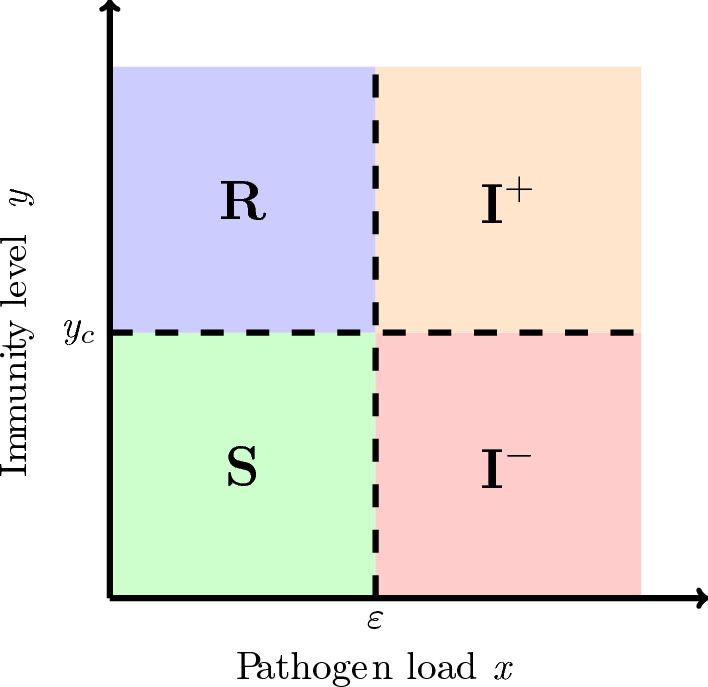
Discrete partition of the immune-pathogen state space in the (*x*, *y*) plane. The dashed lines represent the infection threshold $\varepsilon$ and the immune protection threshold $y_c$, separating compartments: susceptible (S), infected with low immunity ($I^-$), infected with high immunity ($I^+$), and recovered (R).

This classification provides a bridge between continuous immune-pathogen dynamics and discrete epidemiological compartments, facilitating trait-structured population-level analysis.(See Figure 2).

To write the population-level model, we define compartment-specific indicator functions based on compartment classification thresholds:

3.1 $$\begin{aligned} S(t; y_0)&= \textbf{1}_{\{x(t; y_0) < \varepsilon \}} \cdot \textbf{1}_{\{y(t; y_0) \le y_c\}}, \quad S(t) = \int _{y_{\min }}^{y_c} S(t; y_0)\, p(y_0)\,dy_0, \end{aligned}$$

3.2 $$\begin{aligned} R(t; y_0)&= \textbf{1}_{\{x(t; y_0) < \varepsilon \}} \cdot \textbf{1}_{\{y(t; y_0) > y_c\}}, \quad R(t) = \int _{y_{\min }}^{y_c} R(t; y_0)\, p(y_0)\,dy_0, \end{aligned}$$

3.3 $$\begin{aligned} I^-(t; y_0)&= \textbf{1}_{\{x(t; y_0) \ge \varepsilon \}} \cdot \textbf{1}_{\{y(t; y_0) \le y_c\}}, \quad I^-(t) = \int _{y_{\min }}^{y_c} I^-(t; y_0)\, p(y_0)\,dy_0, \end{aligned}$$

3.4 $$\begin{aligned} I^+(t; y_0)&= \textbf{1}_{\{x(t; y_0) \ge \varepsilon \}} \cdot \textbf{1}_{\{y(t; y_0) > y_c\}}, \quad I^+(t) = \int _{y_{\min }}^{y_c} I^+(t; y_0)\, p(y_0)\,dy_0, \end{aligned}$$

where $\textbf{1}_{A}$ is the indicator function of the set *A*. This projection framework enables a direct link between individual immune-pathogen trajectories and population-level epidemiological indicators. Unlike traditional SIR models based on phenomenological rates, this approach derives compartmental dynamics from mechanistic within-host rules. It also allows grouping individuals by innate trait, thereby identifying which host classes are most vulnerable or most resistant.

The compartmental indicators defined above rely on strict threshold conditions for both pathogen load and immune level. While useful for classification, this approach imposes sharp transitions that may not reflect biological or clinical host responses. In particular, both detectability of infection and the delineation between low and high immunity can be gradual, context-dependent, and affected by measurement uncertainty. To address this, we introduce below (see Remark 5.1) a regularized projection framework in which the discrete indicator functions are replaced by continuous, differentiable switching functions (used for numerical simulations and visualization).

## Basic Reproduction Number $\mathcal {R}_0$ under Re-Exposure Dynamics

### Next-generation operator and definition of ${\mathcal {R}}_0$

We compute the basic reproduction number $\mathcal {R}_0$ using the spectral approach developed in Thieme (2009b), here adapted to a host-trait structured formulation of environmental re-exposure. We also consider the explicit form of $\beta$ defined as in Example 2.3. We observe that the pathogen-free equilibrium is given by $E_0 = (0, y_0)$.

Linearizing the system (2.1) around $E_0 = (0, y_0)$, we obtain:

4.1 $$\begin{aligned} \frac{\partial x(t; y_0)}{\partial t} = - (\delta y_0 + r) x(t; y_0) + \frac{\sigma d \beta _0}{b} e^{-a y_0} \int _{y_{\min }}^{y_c} \widehat{w}(y_0, \tilde{y}_0) e^{-\theta \tilde{y}_0} x(t; \tilde{y}_0) p(\tilde{y}_0) \, d\tilde{y}_0, \end{aligned}$$

where the normalized kernel is defined by

4.2 $$\begin{aligned} \widehat{w}(y_0, \tilde{y}_0) = \frac{w(y_0, \tilde{y}_0)}{\displaystyle \int _{y_{\min }}^{y_c} w(y_0, z) p(z) \, dz}. \end{aligned}$$

We define the linear operator $\mathcal {A}: L^1(y_{\min }, y_c) \rightarrow L^1(y_{\min }, y_c)$ by:

4.3 $$\begin{aligned} (\mathcal {A} u)(y_0) = - (\delta y_0 + r) u(y_0) + \frac{\sigma d \beta _0}{b} e^{-a y_0} \int _{y_{\min }}^{y_c} \widehat{w}(y_0, \tilde{y}_0) e^{-\theta \tilde{y}_0} u(\tilde{y}_0) p(\tilde{y}_0) \, d\tilde{y}_0. \end{aligned}$$

Next, we decompose $\mathcal {A} = B + C$, where:

4.4 $$\begin{aligned} (Bu)(y_0)&= - (\delta y_0 + r) u(y_0), \quad D(B) = L^1(y_{\min }, y_c), \end{aligned}$$

4.5 $$\begin{aligned} (Cu)(y_0)&= \frac{\sigma d \beta _0}{b} e^{-a y_0} \int _{y_{\min }}^{y_c} \widehat{w}(y_0, \tilde{y}_0) e^{-\theta \tilde{y}_0} u(\tilde{y}_0) p(\tilde{y}_0) \, d\tilde{y}_0, \quad D(C) = L^1(y_{\min }, y_c). \end{aligned}$$

The operator *B* generates a positive analytic semigroup on $L^1(y_{\min }, y_c)$, given by:

4.6 $$\begin{aligned} [T(t)u](y_0) = e^{-(\delta y_0 + r)t} u(y_0). \end{aligned}$$

Its spectral bound satisfies $s(B)=-(\delta y_{\min }+r)<0$, and the resolvent of *B* reads

4.7 $$\begin{aligned} [(\lambda - B)^{-1}u](y_0) = \frac{u(y_0)}{\lambda + \delta y_0 + r}, \quad \lambda \ge 0. \end{aligned}$$

Now, we define the next-generation operator by

4.8 $$\begin{aligned} \mathbb {K}_\lambda := C(\lambda - B)^{-1}, \quad \text {in particular} \quad \mathbb {K}_0 = C(- B)^{-1}. \end{aligned}$$

Biologically, $\mathbb {K}_0$ maps a distribution of currently infected hosts onto the distribution of new infections they generate over one full infectious period, accounting for host-trait heterogeneity in re-exposure.

The basic reproduction number is defined as:

4.9 $$\begin{aligned} \mathcal {R}_0 := \widehat{\rho }(\mathbb {K}_0), \end{aligned}$$

where $\widehat{\rho }(\cdot )$ denotes the spectral radius.

Stability of the pathogen-free equilibrium: According to Theorems 3.16 and 3.17 in Thieme (2009b), the pathogen-free equilibrium $E_0$ is locally asymptotically stable if $\mathcal {R}_0<1$ and unstable if $\mathcal {R}_0>1$.

### Global Mixing Case ($w \equiv 1$)

When $w \equiv 1$, the normalized kernel satisfies $\widehat{w}(y,z) \equiv 1.$ In this case the integrand becomes separable, and the $(n+1)$-fold iteration simplifies. Defining

4.10 $$\begin{aligned} R(\lambda ) := \frac{\sigma d \beta _0}{b} \int _{y_{\min }}^{y_c} \frac{e^{-(a+\theta ) z}}{\lambda + \delta z + r} p(z) \, dz, \end{aligned}$$

we obtain

4.11 $$\begin{aligned} [(C(\lambda - B)^{-1})^{n+1} u](y_0) = \frac{\sigma d \beta _0}{b} e^{-a y_0} \, R(\lambda )^n \int _{y_{\min }}^{y_c} \frac{e^{-(a+\theta ) z}}{\lambda + \delta z + r} u(z) p(z) \, dz. \end{aligned}$$

Hence the basic reproduction number is

4.12 $$\begin{aligned} \mathcal {R}_0 = R(0) = \frac{\sigma d \beta _0}{b} \int _{y_{\min }}^{y_c} \frac{e^{-(a+\theta ) z}}{\delta z + r} p(z) \, dz. \end{aligned}$$

To provide more insights about the relevance of the basic reproduction number in our setting we introduce the following definitions.

#### Definition 4.1

*(Trait-specific reproduction number under re-exposure)* Consider the pathogen-free equilibrium $E_0$. For a host with baseline immune trait $y_0$, we define the trait-specific reproduction number

4.13 $$\begin{aligned} \mathcal {R}_0(y_0) := \underbrace{\sigma }_{\begin{array}{c} \text {exposure}\\ \text {frequency} \end{array}} \;\times \; \underbrace{F'(0)}_{\begin{array}{c} \text {marginal exposure gain}\\ \text {at low population pressure} \end{array}} \;\times \; \underbrace{e^{-ay_0}}_{\begin{array}{c} \text {probability of establishment}\\ \text {(effective uptake) at }y=y_0 \end{array}} \;\times \; \underbrace{\frac{1}{r+\delta y_0}}_{\begin{array}{c} \text {mean within-host lifetime}\\ \text {near the pathogen-free equilibrium} \end{array}}. \end{aligned}$$

Here, $F(\bar{z})=\frac{d\bar{z}}{b+\bar{z}}$, with $\bar{z}:=\bar{z}(x,y)=\int _{y_{\min }}^{y_c} \beta (x(t; z), y(t; z))\, p(z)\, dz$ and $\beta$ defined in (2.8). Hence, $F'(0):= \left. \frac{\partial F}{\partial x}\right| _{(x=0, y=y_0)}=\beta _0\frac{d}{b}e^{-\theta y_0}$.

#### Definition 4.2

*(Population-level basic reproduction number under re-exposure)* The *population-level* basic reproduction number of model (2.1) is defined as the trait-average

4.14 $$\begin{aligned} \mathcal {R}_0 := \int _{y_{\min }}^{y_c}{\mathcal {R}}_0(z)\,p(z)\,dz. \end{aligned}$$

The rigorous derivation of (4.14) from the linearization at the pathogen-free equilibrium is given in (4.12). Now, we provide an interpretation of our basic reproduction number.

**Biological interpretation**. When $w\equiv 1$, the re-exposure is governed by the population-level pathogen pressure $L_\beta$ defined in (2.7), which combines pathogen shedding across hosts and feeds back instantaneously into individual exposure; no additional dynamical compartment is introduced. Accordingly, ${\mathcal {R}}_0(y_0)$ admits the standard contact $\times$ establishment $\times$ duration interpretation near the pathogen-free equilibrium: (i) $\sigma F'(0)=\sigma \beta _0 d/be^{-\theta y_0}$ is the initial re-exposure gain at low pathogen pressure; (ii) $e^{-ay_0}$ is the probability of establishment (effective uptake) upon exposure at the pathogen-free immune state $y=y_0$; and (iii) $(r+\delta y_0)^{-1}$ is the mean within-host lifetime of the pathogen near the pathogen-free equilibrium. Indeed, for small pathogen loads the linearized within-host dynamics satisfy the equation (4.1), so that pathogen load decays at rate $r+\delta y_0$; its inverse provides the characteristic time scale over which the pathogen remains present in the host following a small successful exposure. Because shedding is function of the pathogen load, this lifetime also corresponds to the effective duration over which the host contributes to $L_\beta$ during the early stage of infection.

Consequently, ${\mathcal {R}}_0(y_0)$ can be interpreted as the expected number of new effective infection events generated by a single newly infected host of trait $y_0$ introduced into an otherwise pathogen-free population, during the period in which its within-host pathogen load remains close to the pathogen-free equilibrium. Averaging over the trait distribution yields the population-level basic reproduction number ${\mathcal {R}}_0$ in Definition 4.2.

In particular, ${\mathcal {R}}_0$ provides a local invasion threshold for the pathogen-free equilibrium: ${\mathcal {R}}_0>1$ implies initial growth of small introductions near the pathogen-free state. Conversely, ${\mathcal {R}}_0<1$ ensures local stability of the pathogen-free equilibrium but does not preclude coexistence with other attractors (e.g., chronic/endemic states) away from the pathogen-free state.

A complete characterization of the long-time dynamics of the coupled system with re-exposure remains open. In particular, we do not currently have a global stability result for the disease-free equilibrium when ${\mathcal {R}}_0<1$. Numerical simulations suggest that infection may persist in the population even for ${\mathcal {R}}_0<1$ (see Section 5.2), indicating that ${\mathcal {R}}_0$ may not provide a sharp threshold in this setting. These observations also motivate a mathematically rigorous study of the possible occurrence of backward bifurcation and related multistability phenomena in the coupled model. A rigorous analysis of the global dynamics, including conditions for convergence to the disease-free equilibrium, mechanisms leading to persistence, and the characterization of any backward bifurcation structure, will be presented in forthcoming work.

### Effect of the Trait-Mixing Kernel on the Basic Reproduction Number

We investigate how the mixing bandwidth $\sigma _w$ affects the spectral radius of the next-generation operator $\mathbb {K}_0:= C(-B)^{-1}$ defined in (4.8). Using the Gaussian family $w_{\sigma _w}$ (and its *p*-weighted normalization ${\widehat{w}}_{\sigma _w}$), we denote the corresponding operator by ${\mathbb {K}}_{\sigma _w}$ and set

$$ {\mathcal {R}}_0(\sigma _w) := \widehat{\rho }({\mathbb {K}}_{\sigma _w}). $$

Since ${\widehat{w}}_{\sigma _w}$ is bounded and continuous on the compact set $[y_{\min },y_c]^2$, ${\mathbb {K}}_{\sigma _w}$ is a positive compact operator on $C([y_{\min },y_c])$; we work in this space for the kernel-limit analysis.

Throughout this section, assume $p \in C([y_{\min },y_c])$ satisfies $p \ge p_{\min } > 0$ and $\int _{y_{\min }}^{y_c} p(z)\,dz = 1$. Let

$$ k_{\sigma _w}(y,z) := {\widehat{w}}_{\sigma _w}(y,z)p(z), \qquad \text {so that} \quad \int _{y_{\min }}^{y_c} k_{\sigma _w}(y,z) \, dz = 1, $$

and rewrite

$$ ({\mathbb {K}}_{\sigma _w}u)(y) = \frac{\sigma d\beta _0}{b} e^{-ay} \int _{y_{\min }}^{y_c} \frac{e^{-\theta z}}{\delta z+r} \, u(z)\, k_{\sigma _w}(y,z) \, dz. $$

Using Collatz–Wielandt (Anselone and Lee 1974, Theorem 6.1), for any $u\in C([y_{\min },y_c])$ with $u>0$,

4.15 $$\begin{aligned} \sup _{u>0}\ \inf _{y\in [y_{\min },y_c]}\ \frac{({\mathbb {K}}_{\sigma _w}u)(y)}{u(y)} \ \le \ \widehat{\rho }({\mathbb {K}}_{\sigma _w}) \ \le \ \inf _{u>0}\ \sup _{y\in [y_{\min },y_c]}\ \frac{({\mathbb {K}}_{\sigma _w}u)(y)}{u(y)}. \end{aligned}$$

By choosing $u(z)=e^{-az}>0$ in (4.15), we obtain that

4.16 $$\begin{aligned} \inf _{y \in [y_{\min },y_c]} \Phi _{\sigma _w}(y) \le {\mathcal {R}}_0(\sigma _w) \le \sup _{y \in [y_{\min },y_c]} \Phi _{\sigma _w}(y), \end{aligned}$$

where $$\displaystyle \Phi _{\sigma _w}(y):=\frac{\sigma d \beta _0}{b} \int _{y_{\min }}^{y_c}\frac{e^{-(a+\theta ) z}}{\delta z+r} k_{\sigma _w}(y, z)dz.$$

Next, choosing $u(z) = e^{\theta z}(\delta z + r)$ in (4.15) gives the upper bound

4.17 $$\begin{aligned} \inf _{y\in [y_{\min },y_c]} \frac{\sigma d\beta _0}{b}\frac{e^{-(a+\theta )y}}{\delta y + r}\le {\mathcal {R}}_0(\sigma _w)\le \sup _{y\in [y_{\min },y_c]} \frac{\sigma d\beta _0}{b}\frac{e^{-(a+\theta )y}}{\delta y + r}. \end{aligned}$$

We thus have the following result.

#### Proposition 4.3

Let

$$ {\mathcal {R}}_0^{\textrm{loc}}:=\sup _{y\in [y_{\min },y_c]} \frac{\sigma d\beta _0}{b}\frac{e^{-(a+\theta )y}}{\delta y + r}, \qquad {\mathcal {R}}_0^{\textrm{glob}}:=\frac{\sigma d\beta _0}{b}\int _{y_{\min }}^{y_c} \frac{e^{-(a+\theta )z}}{\delta z+r}p(z)\,dz. $$

Then:

(i): $$\displaystyle \limsup _{\sigma _w\rightarrow 0}{\mathcal {R}}_0(\sigma _w)\le {\mathcal {R}}_0^{\textrm{loc}}.$$
(ii): As $\sigma _w\rightarrow \infty$ (global mixing), ${\mathcal {R}}_0(\sigma _w)\rightarrow {\mathcal {R}}_0^{\textrm{glob}}$.

#### Proof

The point (*i*) follows directly from (4.17).

As $\sigma _w\rightarrow \infty$, one has ${\widehat{w}}_{\sigma _w}(y,z)\rightarrow 1$ uniformly on $[y_{\min },y_c]^2$ and thus $k_{\sigma _w}(y,z)\rightarrow p(z)$ uniformly; hence $\Phi _{\sigma _w}$ converges uniformly in *y* to ${\mathcal {R}}_0^{\textrm{glob}}$. Taking the limit in (4.16) as $\sigma _w\rightarrow \infty$ gives (*ii*). $\square$

## Simulation Experiments

### Combined Effect of Inoculum Size and Immune Trait Under Nonlinear Thresholds

**Fig. 3:**
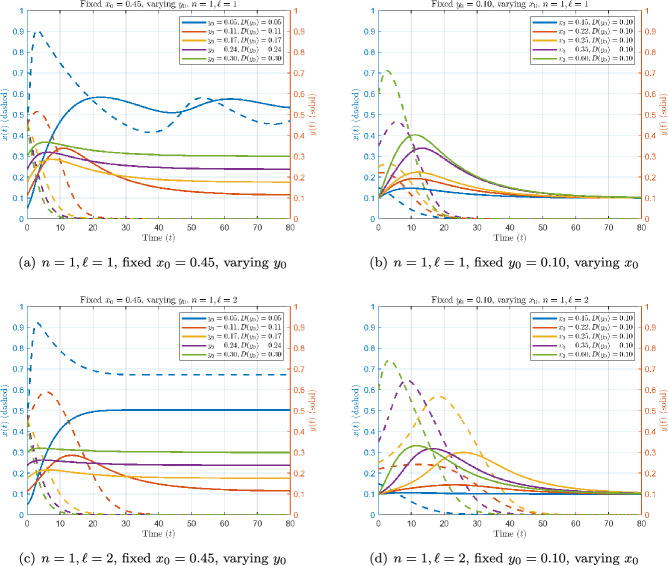
Dynamics with linear threshold $n=1$ and feedback exponent $\ell =1$ (top) vs. $\ell =2$ (bottom). Dashed: pathogen load *x*(*t*); solid: immune response *y*(*t*).

To understand the distinct contributions of pathogen inoculum and host immune heterogeneity to infection dynamics, we first consider a simplified setting where re-exposure is absent. Specifically, we set $\rho (t; y, y_0) = 0,$ thereby isolating the contribution of the environmental feedback in the within-host system. We then investigate the joint impact of the initial pathogen load $x_0$ and the baseline immune level $y_0$ on within-host infection dynamics, under various threshold nonlinearities. These are modeled by the dose-threshold function defined in (2.2), which specifies the minimum pathogen density required for self-sustained growth. The exponent *n* controls how sharply the threshold increases with $y_0$. We performed simulations of the within-host model (2.1) under two setups:

(i) fixed inoculum size $x_0 = 0.45$, varying $y_0 \in \{0.05,\,0.11,\,0.15,\,0.20,\,0.25,\,0.30\}$;
(ii) fixed immune trait $y_0 = 0.10$, varying $x_0 \in \{0.08,\,0.20,\,0.30,\,0.45,\,0.60\}$.

Unless stated otherwise, parameters are $r = 0.111$, $K = 1$, $\delta = 0.9$, $\eta = 0.09$, $\kappa _0 = 0.8$, $c = 0.8$, $D_{\min }=0.05$, $D_{\max }=0.30$, $y_{\min }=0.05$, $y_c = 0.30$.

Case 1-2: Linear threshold ($n=1$) with linear vs. nonlinear feedback ($\ell =1,2$).

For $n=1$, the threshold $D(y_0)$ scales linearly with baseline immunity, so clearance becomes progressively easier as $y_0$ increases. In Figures 3a and 3c , infections are cleared rapidly for $y_0 \ge 0.11$, whereas $y_0=0.05$ sustains a pathogen steady state. Comparing Figure 3b with Figure 3d , raising $\ell$ from 1 to 2 changes the activation term from $\kappa _0 x^1$ to $\kappa _0 x^2$: recruitment is weak when *x* is small, since in our simulations $x<1$ and thus $x^2<x$, which delays the immune action. As a result, the pathogen load *x*(*t*) reaches a higher peak, occurring later in time, for $\ell =2$ compared to $\ell =1$. The immune response *y*(*t*) is also delayed; in most cases its peak is lower in case $\ell =2$, except for some intermediate $y_0$ values (e.g. the yellow curve) where a higher peak occurs. Even when the peak is smaller, the response tends to persist longer, so that the cumulative immune action is greater. Overall, clearance is slower than under $\ell =1$.

Case 3-4: Nonlinear threshold ($n=2$) with linear vs. nonlinear feedback ($\ell =1,2$).

**Fig. 4:**
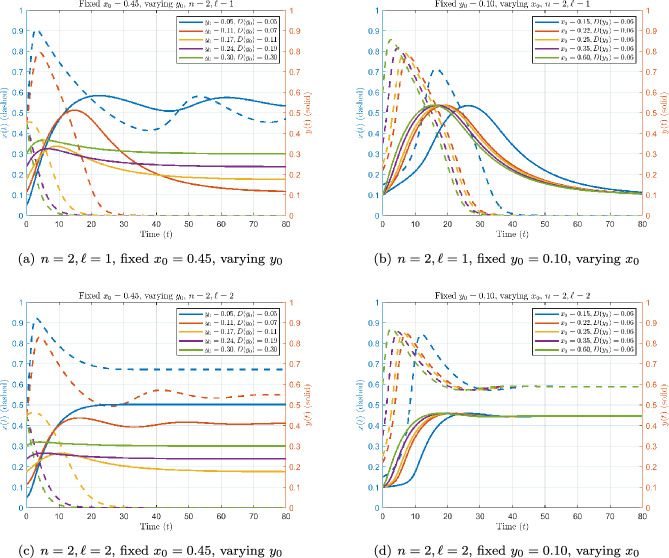
Dynamics with nonlinear threshold $n=2$ and feedback exponent $\ell =1$ (top) vs. $\ell =2$ (bottom).

With $n=2$, the threshold $D(y_0)$ increases quadratically with baseline immunity. Biologically, this means that a host with low $y_0$ faces a disproportionately high barrier for pathogen self-sustainment: a small increase in $y_0$ has little effect until $y_0$ approaches intermediate values, at which point clearance improves abruptly. In Figures 4a and 4c, for $x_0=0.45$, a low $y_0$ value (0.05 in both panels, and 0.11 only in Figure 4c) sustains a high pathogen steady state, whereas a higher $y_0$ enables more effective pathogen clearance.

The feedback exponent further modulates outcomes. Under $\ell =1$, recruitment is proportional to *x*, leading to slow immune dynamics. Under $\ell =2$, recruitment scales as $x^2$, which weakens immune response at low pathogen loads. As a result, the pathogen can initially grow larger, as the immune action is delayed. Clearance, when it occurs, is slower than for $\ell =1$, and alternative outcomes appear: depending on initial conditions, trajectories may either clear or converge to a chronic positive equilibrium. In Figures 4b and 4d, with $y_0=0.10$, all inocula clear under $\ell =1$ (Figure 4b), but under $\ell =2$ (Figure 4d), all tested inocula converge to a nonzero steady state, so that infection persists and clearance does not occur at this baseline immunity. Across all scenarios, clearance is the dominant outcome when baseline immunity is sufficiently strong ($y_0 \gtrsim 0.15$) or when immune feedback is efficient. The parameters *n* and $\ell$ play complementary but distinct roles. The parameter *n* controls how sharply the pathogen growth threshold $D(y_0)$ rises with baseline immunity. Larger values of *n* disproportionately penalize hosts with low $y_0$, making clearance more difficult when immunity is weak. In contrast, $\ell$ regulates how sensitively immune activation responds to pathogen density. Larger values of $\ell$ delay immune activation at low *x*, allowing the pathogen to grow to higher levels before the response sets in.

Linear configurations ($n=1$ or $\ell =1$) mostly lead to robust clearance across tested ranges. In contrast, the nonlinear combination $(n,\ell )=(2,2)$ yields chronic infection at low $y_0$, while higher $y_0$ ensures clearance. For intermediate immunity levels the outcome depends sensitively on $y_0$, creating a threshold-like separation between clearance and persistence. At $y_0=0.10$, however, all tested inocula converge to chronic equilibria. Biologically, this shows how the interaction between a steep threshold and cooperative immune activation can generate either robust clearance or long-term persistence, depending on baseline immunity.(See Table 2).

**Table 2:** Summary of infection outcomes across parameter sets $(n,\ell )$. Linear cases mainly yield clearance on the tested range; the nonlinear combination (2, 2) generates qualitatively distinct outcomes (chronicity, multiple equilibria)

**Setup**	$(n,\ell )$	**Observation on tested range**
$x_0=0.45$, $y_0\uparrow$	(1, 1)	Clearance for $y_0 \ge 0.11$; high steady state at $y_0=0.05$
$y_0=0.10$, $x_0\uparrow$	(1, 1)	All inocula clear; larger $x_0 \Rightarrow$ higher peaks
$x_0=0.45$, $y_0\uparrow$	(1, 2)	Slower suppression than (1, 1); all but smallest $y_0$ clear
$y_0=0.10$, $x_0\uparrow$	(1, 2)	All inocula clear; larger $x_0$ gives later and higher *x* peaks; *y* peaks are delayed and usually lower, except at some intermediate $y_0$.
$x_0=0.45$, $y_0\uparrow$	(2, 1)	Chronic for $y_0=0.05,0.11$; clearance for $y_0 \ge 0.15$
$y_0=0.10$, $x_0\uparrow$	(2, 1)	All inocula clear, clearance times vary with $x_0$
$x_0=0.45$, $y_0\uparrow$	(2, 2)	Low $y_0$ (0.05, 0.11): persistent steady states; higher $y_0$: clearance
$y_0=0.10$, $x_0\uparrow$	(2, 2)	All inocula converge to a positive steady state (chronic infection); clearance does not occur in the tested range.

### Simulated Immune-Pathogen Trajectories and Their Projection onto *S*, $I^-$, $I^+$, and *R* States

We illustrate the projection procedure of Section 3 in a heterogeneous host population of 200 individuals. Baseline immune traits are sampled as $y_0 \sim \textrm{Beta}(2,8)$, rescaled to $[y_{\min }=0.05,\,y_c=0.2]$, such that most hosts lie below the protection threshold $y_c$. Infection is seeded in a single host with the lowest immunity $y_0=0.0504$ and with a pathogen load $x_0(y_0)=0.4675.$

#### Remark 5.1

*(Regularization used in numerical simulations)* We mention that the projection method used in Section 3 is defined using indicator functions, which introduce discontinuities. For numerical simulations and for plotting purposes, we replace each indicator by a smooth sigmoid approximation. Specifically, we use

$$\begin{aligned} \textbf{1}_{\{x<\varepsilon \}} \approx \frac{1}{1+\exp \!\big (k_x(x-\varepsilon )\big )}, \qquad \textbf{1}_{\{x\ge \varepsilon \}}\approx \frac{1}{1+\exp \!\big (-k_x(x-\varepsilon )\big )}, \end{aligned}$$

$$\begin{aligned} \textbf{1}_{\{y\le y_c\}}\approx \frac{1}{1+\exp \!\big (k_y(y-y_c)\big )}, \qquad \textbf{1}_{\{y> y_c\}}\approx \frac{1}{1+\exp \!\big (-k_y(y-y_c)\big )}. \end{aligned}$$

where $k_x, k_y>0$ controls the steepness of the transition. Unless otherwise stated, all simulations use this smooth approximation with $k_x=k_y=100$. In practice, the main effect of this regularization is to smooth out otherwise abrupt transitions, so that the plotted curves become continuous and visually smoother.

Re-exposure kernel. Hosts are exposed to contamination from immunologically similar hosts through a Gaussian kernel in trait space:

$$ w_{\sigma _w}(y_0,z) = \exp \!\Big (-\frac{(y_0-z)^2}{2\sigma _w^2}\Big ), \qquad \sigma _w =0.001, $$

so that the effective pressure for a host with trait $y_0$ is the *p*-weighted average defined in (2.6). This trait-local mixing enforces gradual spread along the trait axis, in contrast to the well-mixed limit $w \equiv 1$.

We set within-host parameters $$(r,K,\delta ,\eta ,\kappa _0,c,a)=(0.111,\,1,\,0.9,\,0.09,$$$$ \,0.8,\,0.8,\,10)$$; re-exposure parameters $$(b,d,\beta _0,K_0)=(2,\,0.2,\,1.6,\,0.05)$$; and thresholds $$(\varepsilon ,y_c)=(0.2,\,0.2)$$. Two re-exposure intensities are compared: $$\sigma =1.7$$ (subcritical) and $$\sigma =3.4$$ (supercritical), yielding numerical reproduction numbers

$$ \mathcal {R}_0 \approx 0.953 \quad \text {and} \quad \mathcal {R}_0 \approx 1.906, $$

under local trait-based mixing. We simulate the cases $n=\ell =1$ and $n=\ell =2$.

#### Immune-Pathogen Trajectories with Linear Thresholds ($n=\ell =1$)

**Fig. 5:**
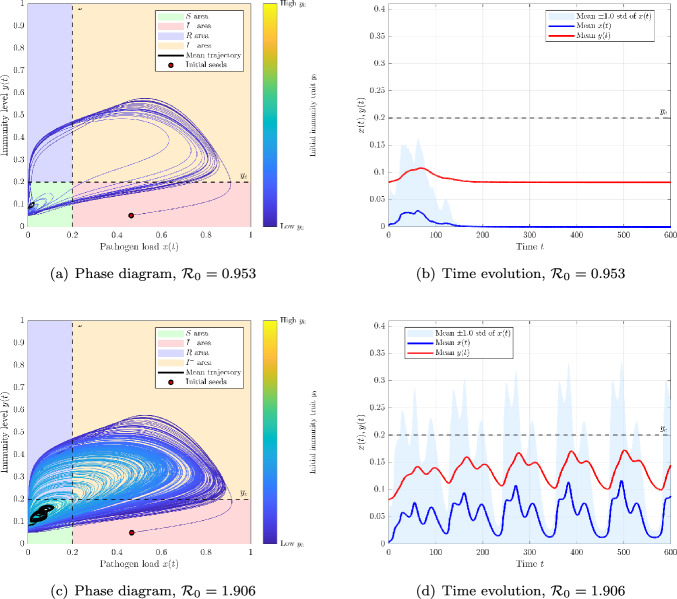
Within-host trajectories under trait-local re-exposure ($\sigma _w=0.001$). **(a,b)** Subcritical case ($\sigma =1.7$, $\mathcal {R}_0=0.953$): a few secondary contaminations occur among immunologically similar hosts, but infection cannot be sustained. **(c,d)** Supercritical case ($\sigma =3.4$, $\mathcal {R}_0=1.906$): infection propagates gradually across the trait axis, crossing pathogen and immune thresholds, and converging to recovery. This illustrates how trait-based proximity drives local cascades, in contrast with global-mixing models

Phase-plane trajectories. Figure 5 shows simulated trajectories in the (*x*, *y*) plane and their time evolution. In the subcritical regime ($\sigma =1.7$, $\mathcal {R}_0=0.953$), pathogen loads rarely cross the infection threshold $\varepsilon$, and most hosts remain susceptible. A few secondary contaminations occur among immunologically similar hosts, but infection quickly fades and the mean trajectory oscillates near baseline. In the supercritical regime ($\sigma =3.4$, $\mathcal {R}_0=1.906$), the seed host exceeds $\varepsilon$, inducing spread across the trait axis: trajectories first rise in *x*, then cross the immune threshold $y_c$, and ultimately converge toward recovery. The mean trajectory captures this ordered progression. Red dots indicate the seed at $t=0$, while colors reflect baseline $y_0$ (from vulnerable to protected).

**Fig. 6:**
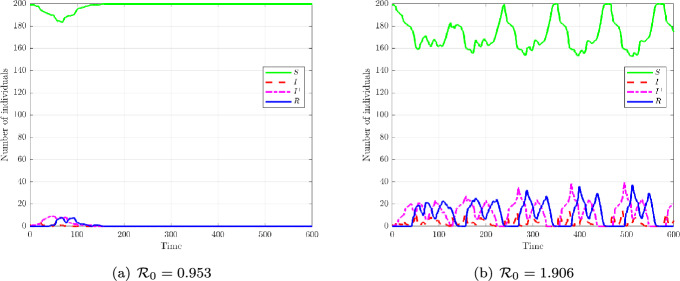
Population-level projections onto $S(t), I^-(t), I^+(t), R(t)$. **(a)** Subcritical regime ($\mathcal {R}_0=0.953$): trait-local re-exposure cannot maintain infection, susceptibles dominate. **(b)** Supercritical regime ($\mathcal {R}_0=1.906$): the cascade $S \rightarrow I^- \rightarrow I^+ \rightarrow R$ emerges directly from intra-host thresholds. This projection ensures consistency across scales without ad hoc couplings.

Population-level projections. Figure 6 displays the smooth compartment curves *S*(*t*), $I^-(t)$, $I^+(t)$, and *R*(*t*). For $\mathcal {R}_0<1$, nearly all hosts remain in *S*, with negligible transitions into $I^-$ or *R*. For $\mathcal {R}_0>1$, a clear cascade emerges: *S* declines, $I^-$ accumulates, then trajectories cross into $I^+$ once $y_c$ is exceeded, and finally converge to *R*.

**Fig. 7:**
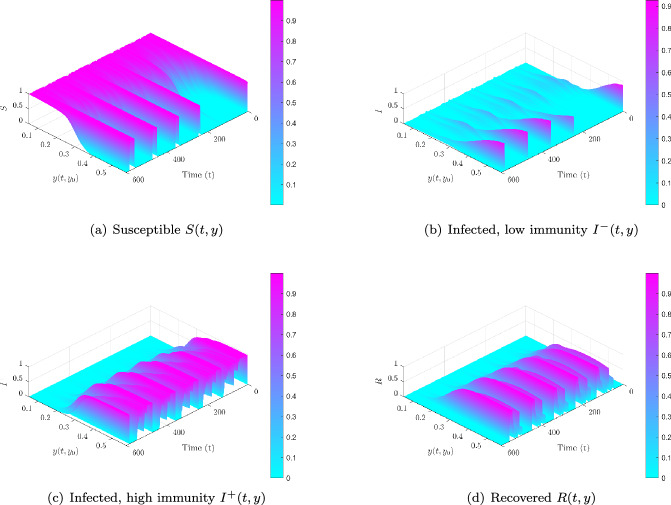
Immune-structured compartment dynamics under supercritical trait-local mixing ($\sigma =3.4$, $\mathcal {R}_0=1.906$). **(a)** *S*(*t*, *y*) concentrated at low *y*, then declines. **(b)** $I^-(t,y)$ peaks near $y\approx 0.15$, marking vulnerable hosts. **(c)** $I^+(t,y)$ emerges close to $y_c=0.2$, reflecting activation of strong immunity. **(d)** *R*(*t*, *y*) accumulates at higher *y*, indicating durable protection. These projections show how heterogeneity and trait-local re-exposure structure infection cascades across immunological profiles

Trait-structured view. Figure 7 resolves compartments by baseline trait $y_0$ in the supercritical case. Initially, *S*(*t*, *y*) is concentrated at low *y*, but these vulnerable hosts are rapidly depleted. $I^-(t,y)$ peaks around $y \approx 0.15$, then shifts upward as $I^+(t,y)$ emerges near $y_c=0.2$. Recovered hosts *R*(*t*, *y*) accumulate progressively at higher *y*, showing how repeated exposures drive the immune trait distribution toward durable protection.

Interpretation. The thresholds $\varepsilon$ and $y_c$ define the infection geometry: $\varepsilon$ separates uninfected from infected, while $y_c$ distinguishes weakly controlled ($I^-$) from strongly controlled ($I^+$) infections. Projection maps intra-host trajectories into clear epidemiological compartments.

This framework is novel compared to previous multi-scale approaches: it avoids ad hoc coupling functions between intra- and inter-host models, where mismatches can arise. Here, the epidemiological states emerge directly from within-host variables and thresholds, ensuring full consistency across scales.

Local mixing plays a decisive role. Under $\mathcal {R}_0<1$, a single infected can induce a handful of nearby contaminations (in trait space), but infection cannot persist and vanishes. Under $\mathcal {R}_0>1$, infection propagates gradually along the trait axis, producing the cascade $S \rightarrow I^- \rightarrow I^+ \rightarrow R$.

#### Immune-Pathogen Trajectories with Quadratic Thresholds ($n=\ell =2$)

We repeat the simulations of Section 5.2.1 with quadratic scaling in both the infection threshold $D(y_0)$ and the immune activation $\kappa (x):=\kappa _0 x^{\ell }=0.8 x^{2}$.

**Fig. 8:**
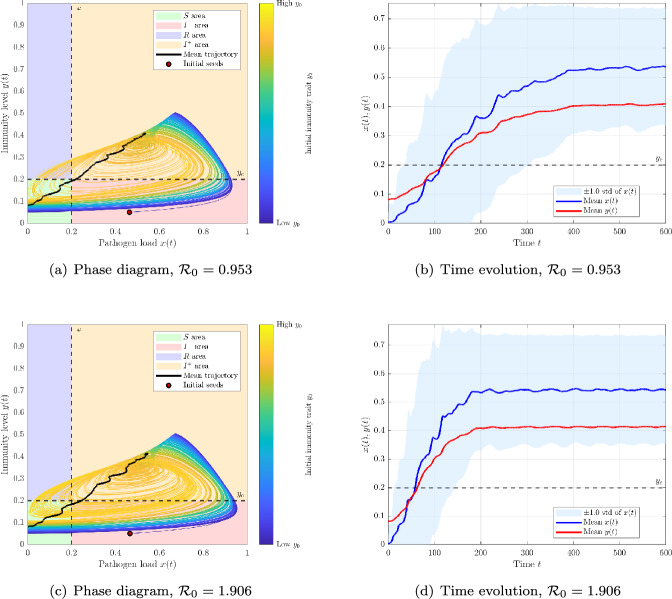
Within-host trajectories under trait-local re-exposure ($\sigma _w=0.001$), quadratic thresholds ($n=\ell =2$). **(a,b)** ${\mathcal {R}}_0=0.953$: coexistence of clearance and chronic infection states depending on initial conditions $(x_0,y_0)$. **(c,d)** ${\mathcal {R}}_0=1.906$: infection spreads across traits with persistent oscillations before stabilization

Phase-plane trajectories. Figures 8 display (*x*, *y*) trajectories for a subcritical case ($\sigma =1.7$, ${\mathcal {R}}_0=0.953$) and a supercritical case ($\sigma =3.4$, ${\mathcal {R}}_0=1.906$). Unlike the linear setting, the system exhibits *bistability*: for ${\mathcal {R}}_0<1$, some trajectories converge to a chronic infection state (positive equilibrium) while others clear, depending on initial conditions $(x_0,y_0)$. For ${\mathcal {R}}_0>1$, the cascade $S \rightarrow I^- \rightarrow I^+ \rightarrow R$ persists, but damped cycles and delayed recoveries are common due to the quadratic recruitment $\kappa _0 x^2$.

**Fig. 9:**
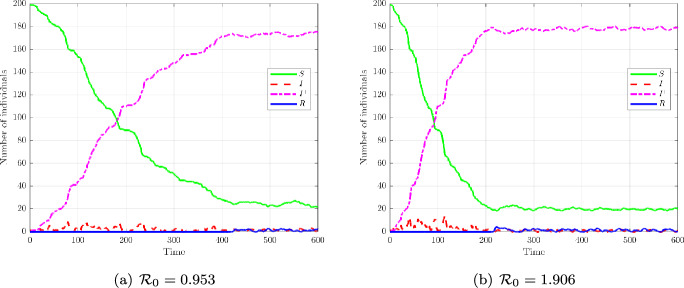
Population-level projections with quadratic thresholds. **(a)** ${\mathcal {R}}_0=0.953$: some hosts remain chronically infected despite ${\mathcal {R}}_0<1$, depending on initial conditions and trait distribution. **(b)** ${\mathcal {R}}_0=1.906$: the cascade $S \rightarrow I^- \rightarrow I^+ \rightarrow R$ occurs, with persistent oscillations

Population-level projections. Figure 9 shows the smooth compartment curves $S(t), I^-(t), I^+(t), R(t)$. For ${\mathcal {R}}_0<1$, the system is not uniformly cleared: a fraction of hosts remain chronically in $I^-/I^+$, maintaining positive pathogen load with small oscillations, a signature of bistability induced by $(n,\ell )=(2,2)$. For ${\mathcal {R}}_0>1$, the cascade $S \rightarrow I^- \rightarrow I^+ \rightarrow R$ emerges, but oscillations persist in all compartments, contrasting with the monotone convergence observed in the linear case.

**Fig. 10:**
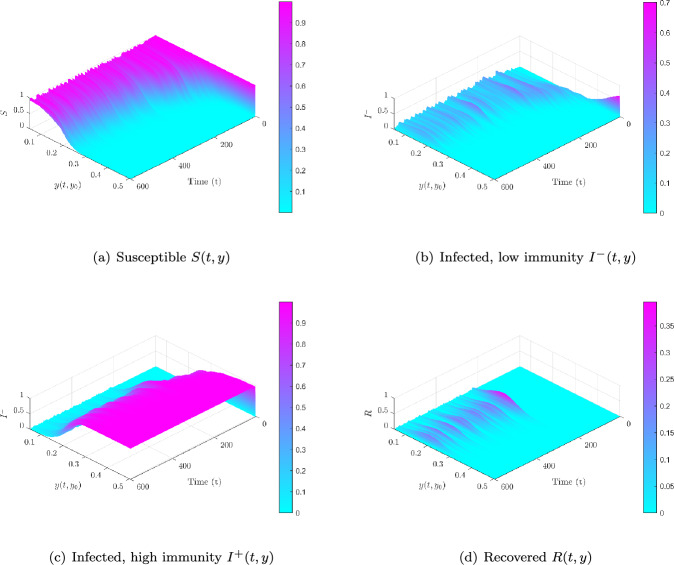
Trait-structured compartment dynamics, supercritical regime ($\sigma =3.4$, ${\mathcal {R}}_0=1.906$), quadratic thresholds. **(a)** *S*(*t*, *y*) concentrated at low *y*, then declines with oscillations. **(b)** $I^-(t,y)$ oscillates around $y\approx 0.15$. **(c)** $I^+(t,y)$ emerges near $y_c=0.2$ and dominates. **(d)** *R*(*t*, *y*) accumulates gradually at higher *y*. Chronic infections also persist for ${\mathcal {R}}_0<1$, driven by the same threshold/recruitment mechanism.

Trait-structured view. Figure 10 resolves compartments by baseline trait $y_0$ in the supercritical regime. Under $(n,\ell )=(2,2)$, *S*(*t*, *y*) is initially concentrated at low $y_0$ and quickly depleted; $I^-(t,y)$ oscillates around $y\approx 0.15$; $I^+(t,y)$ emerges near $y_c=0.2$ and dominates at intermediate times; *R*(*t*, *y*) grows more slowly at higher *y*. The same mechanism explains the persistence of some hosts in $I^\pm$ for ${\mathcal {R}}_0<1$, consistent with the results of Section 5.1 without re-exposure, where chronic equilibria also arise under $(n,\ell )=(2,2)$.

##### Remark 5.2

*(Chronicity vs. backward bifurcation)* With quadratic thresholding $(n,\ell )=(2,2)$, the within-host system does not undergo a backward bifurcation in the strict sense. Instead, it admits chronic infection trajectories: for some initial conditions $(x_0,y_0)$, pathogen load converges to a positive equilibrium rather than clearing. This host-level chronicity becomes epidemiologically relevant in a between-host model, where the coexistence of chronically infected and cleared individuals can produce a population-level backward bifurcation, i.e. an endemic equilibrium coexisting with the disease-free state even when ${\mathcal {R}}_0<1$. This behavior here is only observed in numerical simulations. A mathematically rigorous bifurcation study, including conditions under which a true backward bifurcation and/or multistability occurs in the coupled model, remains open and will be studied in forthcoming work.

### Effect of the Trait-Mixing Kernel width $\sigma _w$ on ${\mathcal {R}}_0$ and Epidemic Persistence

**Fig. 11:**
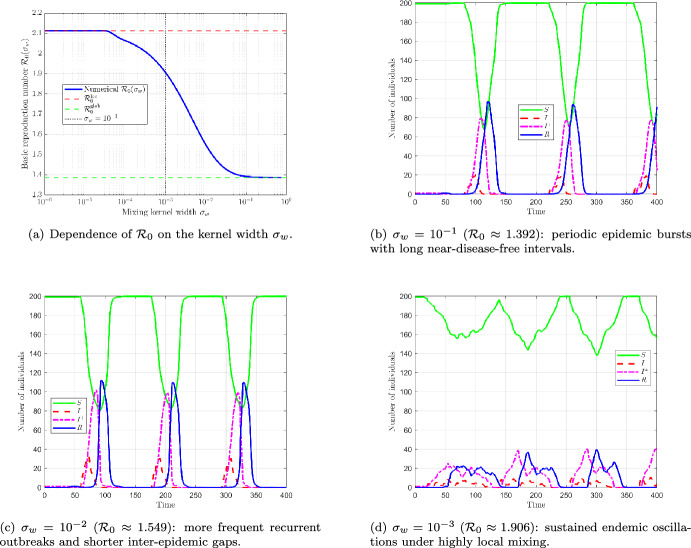
Effect of the trait-mixing kernel width $\sigma _w$ on the reproduction number and epidemic dynamics (Beta-Weak trait distribution, $\sigma =3.4$). **(a)** Numerical values of ${\mathcal {R}}_0(\sigma _w)$ (blue) decrease monotonically as $\sigma _w$ increases, connecting the strongly trait-local regime (small $\sigma _w$) to the effectively global-mixing limit $w\equiv 1$ (green dashed line). The red dashed line indicates an upper bound for ${\mathcal {R}}_0(\sigma _w)$, and the vertical dotted line marks $\sigma _w=10^{-3}$. **(b, c, d)** Smoothed compartment trajectories $(S,I^-,I^+,R)$ for three representative kernel widths.

Figure 11 illustrates how the kernel width $\sigma _w$ influences both ${\mathcal {R}}_0$ and the long-term epidemic dynamics. Figure 11a shows that ${\mathcal {R}}_0(\sigma _w)$ decreases as $\sigma _w$ increases, and converges to the global-mixing value corresponding to $w\equiv 1$. Since the trait interval is relatively narrow, $[y_{\min },y_c]=[0.05,0.2]$ (width 0.15), the choice $\sigma _w=10^{-1}$ already yields a Gaussian kernel that is nearly uniform over the domain. Accordingly, both ${\mathcal {R}}_0$ and the epidemic trajectories are very close to those of the well-mixed limit, with a difference of order $10^{-2}$ in ${\mathcal {R}}_0$.

For $\sigma _w=10^{-1}$, the epidemic is characterized by high and sharp outbreaks of short duration, separated by long low-prevalence periods. As $\sigma _w$ decreases, transmission becomes more concentrated among individuals with similar trait values. In this case, infection spreads more gradually across trait space, from one trait range to nearby ones, so that the epidemic lasts over a longer time interval. This leads to outbreaks that are more frequent, less isolated, and typically less sharply peaked. For the smallest value, $\sigma _w=10^{-3}$, infection remains present over a much larger fraction of time, and the system no longer returns close to the disease-free state, indicating a more persistent regime.

Overall, $\sigma _w$ appears to be a key parameter controlling the transition from nearly global mixing to strongly localized trait-dependent mixing, with important consequences for both ${\mathcal {R}}_0$ and epidemic persistence.

## Global Sensitivity Results

### Numerical Computation of $\mathcal {R}_0$ and Sensitivity Analysis

**Numerical evaluation of** $\mathcal {R}_0$. The basic reproduction number $\mathcal {R}_0$ is defined in Section 2 as the spectral radius of the next-generation operator $\mathbb {K}_0$:

$$\begin{aligned} (\mathbb {K}_0 u)(y_0) = \frac{\sigma \, d\, \beta _0}{b} \, e^{-a y_0} \int _{y_{\min }}^{y_c} \widehat{w}(y_0,z) \, \frac{e^{-\theta z}}{\delta z + r} \, u(z) \, p(z) \, dz , \end{aligned}$$

where $\widehat{w}$ is the row-normalized contact kernel and *p*(*z*) the trait distribution.

Here, the trait distribution is modelled as a mixture of two Beta densities on the interval $(y_{\min },y_c)$:

$$ p(z) = a_3 \, \textrm{Beta}(a_1,a_2) + (1-a_3) \, \textrm{Beta}(a_2,a_1), $$

where $a_1, a_2 > 0$ are shape parameters and $a_3 \in [0,1]$ is the mixing weight, to be chosen later.

Unless otherwise stated, the contact kernel is Gaussian 

$$ w_{\sigma _w}(y_0,z) = \exp \!\Big (-\frac{(y_0-z)^2}{2\sigma _w^2}\Big ), $$

with $\sigma _w >0$ setting the trait-distance bandwidth.

For numerical approximation, the trait interval $[y_{\min },y_c]$ is discretized into $N_y$ uniformly spaced points, integrals are evaluated with the trapezoidal rule, and the corresponding matrix *K* is strictly positive. Its dominant eigenvalue, computed via the power method, provides the approximation $\widehat{\rho }(\mathbb {K}_0)$.

### Sensitivity Analysis Results

The influence of parameters on $\mathcal {R}_0$ is assessed by two complementary approaches: the *Latin Hypercube Sampling* (LHS) with correlation ranking (McKay et al. 2000), and variance-based Sobol indices (Saltelli 2002; Saltelli et al. 2010), namely first-order indices $\textbf{S}$ and total-order indices $\textbf{S}_T$.

We now present the results of this sensitivity analysis of $\mathcal {R}_0$ with respect to model parameters.

**Fig. 12:**
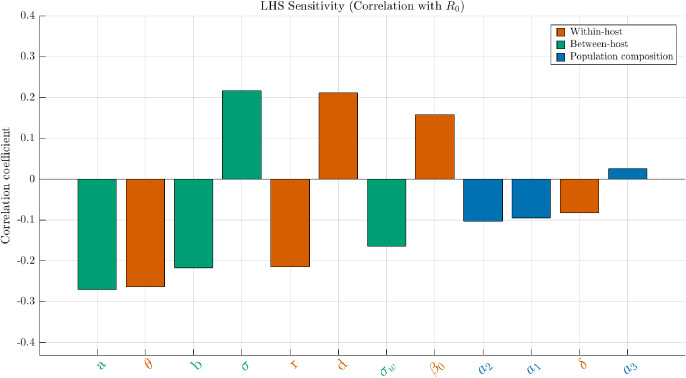
**LHS correlation with** $\mathcal {R}_0$. Parameters on the *x*-axis are ordered from left to right by decreasing absolute value of their correlation coefficient. Colors encode parameter families (color-blind safe palette):  = within-host ($d,\delta ,r,\beta _0,\theta$),  = between-host ($\sigma ,a,\sigma _w,b$),  = population composition ($a_1,a_2,a_3$). Positive bars increase $\mathcal {R}_0$ when the parameter grows; negative bars reduce it. Seed, kernel and ranges match Section 6.1. Parameter definitions are given in Table 1. (Color figure online).

**Fig. 13:**
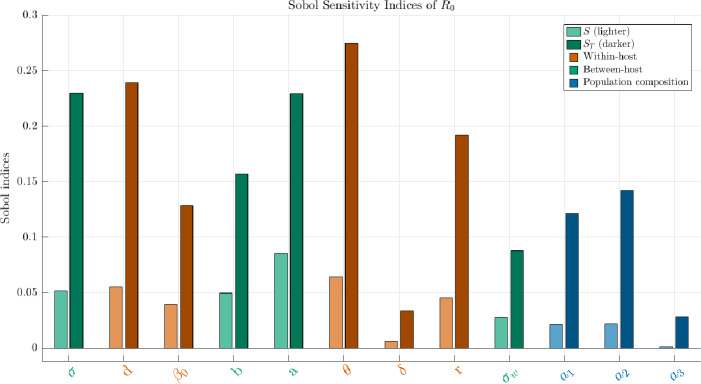
**Sobol variance decomposition of** $\mathcal {R}_0$. For each parameter, the lighter bar is the first-order index $\textbf{S}$ and the darker bar the total index $\textbf{S}_T$. Both share the family color (as in Figure 12). A large gap $\textbf{S}_T-\textbf{S}$ signals strong interactions, meaning the parameter matters mainly through coupling with others.

Within-host parameters (vermillion). In Figure 12, *d* (shedding scale), and $\beta _0$ (baseline shedding) have the strongest positive correlations ($\approx 0.15$-0.25), confirming that higher pathogen output directly raises $\mathcal {R}_0$. Conversely, $\theta$ and *r* are strongly negative. Indeed stronger immune suppression of shedding thereby reducing transmission potential. Moreover, although *r* denotes within-host pathogen growth in (2.1), it plays the role of a decaying rate in the pathogen population in the linearized equation (4.1). The shielding *a* and the immune clearance rate $\delta$ appear with negative signs, consistent with their regulatory effect. In Figure 13, *d*, $\beta _0$ and $\theta$ show $\textbf{S}_T \gg \textbf{S}$, revealing that their influence is amplified mainly via interactions with $\sigma$ (contact intensity).

Between-host structure (bluish-green). $\sigma$ is the dominant positive driver, as expected from its role in re-exposure intensity. Parameter *a* has a strong negative effect and interacts broadly; biologically, this reflects how shielding couples with both contact intensity and shedding. The mixing width $\sigma _w$ has modest additive effects: larger $\sigma _w$ smooths trait-locality, smaller $\sigma _w$ enforces trait-proximity in contacts.

Population composition (navy). $a_1$ (skew toward low-immunity hosts) and $a_2$ (skew toward high-immunity hosts) have weak main effects ($\textbf{S}<0.05$) but large total indices ($\textbf{S}_T$ up to $\approx 0.18$ for $a_1$). This means their role is mostly indirect: composition modulates the effective impact of transmissibility and shielding. Increasing $a_1$ amplifies $\mathcal {R}_0$ by exposing more vulnerable hosts; increasing $a_2$ dampens spread by enriching resistant profiles. $a_3$ contributes negligibly in this setting.

The correlation signs in Figure 12 match the analytic structure of $\mathbb {K}_0$: the term $\sigma d \beta _0/b$ sets transmission gain; $e^{-a y_0}$ and $e^{-\theta z}$ act as trait-based gating terms; $(\delta z+r)^{-1}$ shortens infectious periods. Gaps $\textbf{S}_T-\textbf{S}$ in Figure 13 highlight exactly those parameters that multiply or regulate others in the operator.

Overall, these sensitivity patterns validate the biological interpretation of $\mathcal {R}_0$ in our framework: intra-host processes (*d*, $\beta _0$, $\delta$, $\theta$) dominate the baseline level, while inter-host parameters ($\sigma$, *a*, $\sigma _w$) determine whether local contamination cascades are sustained. Population composition shapes outcomes indirectly through interactions.

## Discussion and Concluding Remarks

We developed a multi-scale model to investigate how immune competence and dose-dependent pathogen exposure shape infection outcomes at both individual and population levels. The model incorporates key immunological mechanisms, including threshold-induced infection, immune boosting, and re-exposure feedback. Our objective was to gain deeper insight into how nonlinear infection thresholds generate sharp transitions between clearance and persistence depending on inoculum size and immune traits, and to extend the within-host framework to the between-host scale through a new projection approach.

Our trait-structured re-exposure mechanism is closely related to the idea of kernel-based approaches in multistrain epidemiology, such as (Gog and Grenfell 2002). In that setting, kernels are used to weight interactions by similarity in strain space, with cross-immunity typically decreasing as strains become more antigenically distant (often described using smooth decays such as Gaussian-type forms). In our case, the same mathematical idea is used, but the source of heterogeneity is different. More precisely, the kernel $w(y_0, z)$ weights interactions by similarity in host immune trait space, so that re-exposure pressure on a host with baseline trait $y_0$ is driven more strongly by shedding from hosts with nearby traits *z*.

Furthermore, a practical advantage of our threshold-based projection is not that it eliminates cross-scale assumptions, but that it reduces the need to specify an independently constructed between-host compartment model with separate transition rates. Instead, the epidemiological classes emerge directly from within-host trajectories through biologically interpretable thresholds. In many multiscale frameworks, within-host variables are coupled to transmission by prescribing an explicit linking function (e.g., viral load to infectiousness or immune response to recovery). This latter relationship is crucial to linking scales but as yet little understood (Gog et al. 2015). Moreover, across existing studies, the assumed functional association varies considerably, and direct empirical support is often limited (Handel and Rohani 2015). Because the choice of linking assumptions can materially affect predictions, different transmission formulations can change $\mathcal {R}_0$ and associated control conclusions (Wonham et al. 2006), and common simplifying assumptions can fundamentally alter epidemiological and evolutionary dynamics (Smith and Ashby 2025). See also the systematic review in Childs et al. (2019), which emphasizes that cross-scale relationships are often complex, nonlinear, and un-intuitive.

**Model without re-exposure** ($\rho =0$). The model without re-infection isolates intrinsic feedbacks between pathogen growth and immune activation, in the absence of external forcing. A central ingredient is the pathogen-density-dependent threshold $D(y_0)$, which formalizes the idea that there is a minimum inoculum required to initiate infection (Yezli and Otter 2011), and that disease outcome depends on the viral dose in the inoculum (Van Damme et al. 2021). This trait-dependent threshold divides the host population, with immune competence ranging from low baseline levels to maximal acquired states and determining both initial susceptibility and long-term immune response trajectories.

Numerical explorations show that infection outcomes are jointly governed by inoculum size $x_0$ and baseline immunity $y_0$. Poorly immunized hosts can sustain infections even for moderate inocula, while well-immunized hosts almost always clear infection. Phase diagrams reveal bistable regions where small changes in $y_0$ or $x_0$ flip outcomes between clearance and persistence. This bistability, generated by a modified value of the Allee-type threshold $D(y_0)$, provides a mechanistic explanation for experimentally observed minimal infectious doses modulated by host heterogeneity (Van Damme et al. 2021).

From a dynamical-systems point of view, these results show that sharp clearance-persistence transitions can emerge purely from internal feedback loops. Immune boosting and waning appear as threshold-driven processes: crossing the activation boundary induces immune amplification, while failure to sustain above it leads to waning of immunity. Overall, outcomes cannot be predicted from inoculum or immunity alone, but only from their nonlinear interplay.

Path to empirical parameterization. Although the model is theoretical, its parameters can be fitted from standard within-host data collected in cohort or challenge studies. The key input is a longitudinal pathogen-load time series *x*(*t*) per host, whose units depend on the system: for viruses, *x*(*t*) may be RT-qPCR viral RNA (copies/mL or Ct values) and/or infectious titre (PFU/mL or $\hbox {TCID}_{50}$/mL); for bacteria, *x*(*t*) is typically quantified by culture as CFU (e.g., *Salmonella* CFU/g of feces). The detection threshold $\varepsilon$ is then fixed by the assay limit of detection and defines when infection/shedding is considered detectable in *x*(*t*).

The immune variable *y*(*t*) represents a level of protection and can be informed either by longitudinal immune measurements when available (e.g., antibody titres, mucosal IgA, cytokine markers, antigen-specific T-cell readouts) or treated as a latent state linked to such correlates. When pathogen and immune readouts are not measured at the same times, the model can still be fitted by treating *y*(*t*) as partially observed and linking measured correlates to *y*(*t*) through an observation model. Baseline heterogeneity is captured by pre-exposure measurements defining $y_0$, from which the empirical distribution $p(y_0)$ is obtained; in our formulation, $y_c$ represents the upper bound of the baseline trait. In practice, it can be estimated from the data as the maximum observed value of $y_0$, or as a high empirical quantile (e.g., the 95th percentile) to make the estimate of $y_c$ less sensitive to outliers. Finally, challenge designs using multiple inoculum doses are especially informative to identify the establishment threshold function $D(y_0)$ across individuals. Parameters can then be estimated by fitting model-predicted trajectories to the observed data, e.g., via nonlinear least-squares or likelihood-based inference under an explicit observation model (Dorešić et al. 2025). Examples of such calibration/validation settings include bacterial challenge studies with quantified shedding (Ivanek et al. 2012), human influenza dose-escalation challenge studies with daily shedding measurements (Han et al. 2019), and avian influenza poultry challenges summarizing shedding levels and duration across experiments (Germeraad et al. 2019); persistent-reservoir identification using combined microbiological testing and immune correlates provides an additional template (Lagos et al. 2024).

**Model with re-exposure** ($\rho \ne 0$). Re-exposure introduces a functional feedback depending on both the population pathogen load and individual immune state. This coupling accounts for direct and indirect transmission, as well as controlled repeated exposures. Mathematically, it leads to a next-generation operator for $\mathcal {R}_0$ structured by trait distributions and mixing kernels, embedding immune thresholds and memory within a unified framework. This goes beyond earlier approaches (Kenne et al. 2023), which relied on ad hoc coupling functions between intra- and inter-host scales.

**Projection onto** *S*, $I^-$, $I^+$, and *R*. We projected heterogeneous within-host trajectories onto discrete compartments using biologically meaningful thresholds $(\varepsilon ,y_c)$. This projection avoids artificial coupling terms and ensures consistency across scales. We have simulated the cases $n=\ell =1$ and $n=\ell =2$. In the linear case ($n=\ell =1$) immune recruitment and thresholds respond smoothly: within-host trajectories follow an ordered progression (rise in *x*, crossing $y_c$, recovery), ${\mathcal {R}}_0<1$ leads to near-uniform clearance with only transient, local contaminations, and the population shows the clean cascade $S\rightarrow I^- \rightarrow I^+ \rightarrow R$ with largely monotone convergence; trait-resolved dynamics show rapid depletion of low-$y_0$ susceptibles, a peak of $I^-$ near $y\!\approx \!0.15$, and progressive accumulation of *R* at higher *y*. By contrast, the quadratic case ($n=\ell =2$) amplifies nonlinearity: immune recruitment is delayed and thresholds are sharper, producing host-level bistability (for some ${\mathcal {R}}_0<1$ initial conditions lead to chronic positive equilibria while others clear), delayed recoveries and damped cycles in individual trajectories, and at the population scale a failure of ${\mathcal {R}}_0<1$ to guarantee clearance (a fraction of hosts may remain chronically infected); when ${\mathcal {R}}_0>1$ the same $S\rightarrow I^- \rightarrow I^+ \rightarrow R$ cascade persists but with persistent oscillations and slower, more heterogeneous movement along the trait axis. In short, increasing nonlinearity from $n=\ell =1$ to $n=\ell =2$ makes outcomes far more sensitive to inoculum size, initial conditions, and trait structure, promoting chronicity, oscillatory dynamics, and the possibility of a population-level backward bifurcation.

**On** $\mathcal {R}_0$ **and backward bifurcations.** In our analytical framework, the basic reproduction number $\mathcal {R}_0$ is obtained by linearization around the disease-free state, and therefore does not depend on the nonlinearity exponent *n* of the dose-threshold function $D(y_0)$, nor on the immune feedback exponent $\ell$ in $\kappa (x)$. As a result, $\mathcal {R}_0$ captures only the local invasion potential at the population level, but ignores the possibility of multiple stable within-host equilibria. Numerical simulations, however, reveal that nonlinear thresholding or feedback can sustain chronic intra-host equilibria, corresponding to persistent infections in subsets of hosts. This discrepancy suggests the occurrence of backward-bifurcation-like behavior: even when $\mathcal {R}_0<1$, infections may persist in the population through chronically infected individuals who fail to clear the pathogen. Such bistability is a well-known feature in epidemic models with strong nonlinearities (Dushoff et al. 1998; Ducrot et al. 2009; Arino et al. Feb. 2012), but its explicit link to within-host chronicity remains underexplored. A rigorous analysis of global stability and bifurcations in this multi-scale framework is therefore a priority for future work, as it would clarify under which conditions standard threshold results based on $\mathcal {R}_0$ may fail to predict eradication.

A final point worth emphasizing is the role of the trait-mixing bandwidth $\sigma _w$ as a bridge between the theoretical and numerical parts of the study. The results shown in Figure 11 are consistent with the framework developed for ${\mathcal {R}}_0(\sigma _w)$: in the parameter regime considered here, more localized trait mixing (smaller $\sigma _w$) is associated with larger values of ${\mathcal {R}}_0$ and more persistent recurrent dynamics, whereas larger $\sigma _w$ leads to behavior closer to the well-mixed limit. In particular, since the trait interval $[y_{\min },y_c]$ is narrow in our simulations, $\sigma _w=10^{-1}$ already yields a nearly uniform Gaussian kernel; accordingly, both ${\mathcal {R}}_0$ and the compartment trajectories are nearly indistinguishable from those of the $w\equiv 1$ case. This highlights that trait-based contact structure influences both invasion potential and the long-term dynamics, including the pace of spread across trait space, the return toward near-disease-free levels, and the occurrence of recurrent outbreaks or sustained oscillations.

### Concluding remarks

Our results demonstrate that immune heterogeneity, nonlinear thresholds, re-exposure feedbacks, and trait-local mixing jointly shape infection dynamics in ways that classical homogeneous models cannot capture. The projection approach integrates within- and between-host scales without ad hoc couplings, ensuring full consistency. Thresholds $(\varepsilon ,y_c)$ provide a geometric link between continuous within-host dynamics and discrete epidemiological states, revealing cascades invisible to traditional SIR models.

In conclusion, our study provides a theoretical foundation for dose-dependent infection dynamics structured by immune competence, and a projection framework that links individual immunity to epidemic outcomes. By integrating re-exposure and heterogeneity, we uncover transitions and cascades invisible to classical models. Future work should calibrate the framework using experimental data and relax the sharp-threshold assumptions. A full characterization of the long-time dynamics, including rigorous global stability results and the possible occurrence of backward bifurcation, remains open and will be addressed in forthcoming work. It should also be interesting to extend the coupling to more complex immune processes, which are the subject of ongoing investigation (Smith and Ashby 2025; Kretzschmar et al. 2022).

Model flexibility. The thresholds $(\varepsilon ,y_c)$ are represented here as fixed and sharp, but their biological meaning is pathogen-dependent and context-specific. For example, $\varepsilon$ denotes the minimal detectable or clinically relevant pathogen load at which a host is considered infected; this level varies widely across diseases and depends on both pathogen biology and diagnostic sensitivity. Similarly, $y_c$ represents an activation level for immune protection, which may differ across pathogens and is often gradual rather than strictly binary. Beyond these illustrative choices, our framework is deliberately general: the distribution of $y_0$ can be adapted to empirical data (e.g., age-structured immunity, prior exposures, genetic variation), and different kernels can be implemented to represent alternative contact structures or pathogen-specific transmission modes. Likewise, while we focused on a reduced (*x*, *y*) system, higher-dimensional within-host models can be projected onto two effective variables (pathogen load and immune competence) before applying the projection.

While we used a scalar immune trait for clarity, the formulation extends naturally to multivariate traits with correlated components, where the immunological dissimilarity $d(y_0,z)$ could be defined via a genetic/phylogenetic metric, allowing the model to capture richer biological structures. This flexibility makes the framework adaptable to diverse pathogens and host populations, while keeping the mathematical analysis tractable.

Nevertheless, the projection approach remains valuable: even with coarse thresholds and reduced dynamics, it generates rich population-level outcomes. Future refinements could relax the sharp-threshold assumption, incorporate longitudinal boosting, or link trait heterogeneity to empirical datasets (e.g. serology, vaccination studies). Such extensions will test the robustness of the cascade $S \rightarrow I^- \rightarrow I^+ \rightarrow R$ and clarify how immune variability controls epidemic thresholds in real populations.

## Data Availability

All numerical results in this article are generated by simulation. The source code and scripts used to generate the figures are available from the authors upon request.
